# Preparation and Characterization of Muscone Oil-Based Cyclodextrin Metal–Organic Frameworks: Molecular Dynamics Simulations and Stability Evaluation

**DOI:** 10.3390/pharmaceutics17040497

**Published:** 2025-04-09

**Authors:** Zifan Qiao, Lihua Chen, Mubarak G. Bello, Shiyu Huang

**Affiliations:** 1 Key Laboratory of Modern Preparation of TCM, Ministry of Education, Jiangxi University of Chinese Medicine, No. 1688, Meiling Road, Nanchang 330004, China; qzf4901@163.com (Z.Q.); mgbello97@gmail.com (M.G.B.); 2 Department of Pharmaceutics and Industrial Pharmacy, Kaduna State University, Kaduna 800244, Nigeria; 3 School of Pharmacy, Jiangxi Science and Technology Normal University, Nanchang 330013, China; syhuang98@126.com

**Keywords:** muscone, γ-cyclodextrin, metal–organic framework, molecular modeling, stability

## Abstract

**Objective:** Muscone (MUS), a primary active component of musk, is known for its significant pharmacological properties. However, its clinical application is limited due to poor water solubility and moderate stability. This study aims to address these limitations by encapsulating MUS within biodegradable γ-cyclodextrin metal–organic frameworks (γ-CD-MOFs) using a solvent-free method to enable oral MUS delivery by improving solubility and stability, pending in vivo validation. **Methods:** MUS was encapsulated into γ-CD-MOFs using a solvent-free method, achieving an optimal loading rate of 10.6 ± 0.7%. Comprehensive characterization was performed using scanning electron microscopy (SEM), X-ray diffraction (XRD), Fourier-transform infrared spectroscopy (FTIR), and thermogravimetric analysis (TGA). Biocompatibility was assessed using RAW264.7 cells, and molecular dynamics simulations were conducted to study the interactions between MUS and γ-CD-MOFs. **Results:** Characterization techniques confirmed the successful encapsulation of MUS into γ-CD-MOFs. Biocompatibility studies revealed no cytotoxicity, indicating that the system is safe for drug delivery. Molecular dynamics simulations showed that MUS preferentially occupies the large spherical cages of γ-CD-MOFs, driven by non-covalent interactions. Solubility tests and in vitro release studies demonstrated that the solubility of MUS was improved after encapsulation within γ-CD-MOFs. Stability assessments indicated that γ-CD-MOFs significantly enhanced the thermal and photostability of MUS, with high residual amounts remaining under various storage conditions. **Conclusions:** This study demonstrates the potential of γ-CD-MOFs to solidify MUS, enhance its solubility, and improve its storage stability, providing a foundation for its future use in pharmaceutical applications.

## 1. Introduction

**Figure 1:**
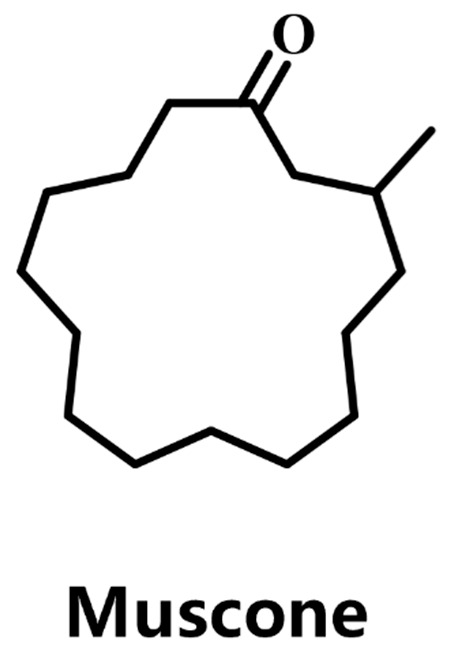
Chemical structure of MUS.

Muscone (MUS), with a chemical structure recognized as 3-methylcyclpentadecanone (Figure 1), is considered the primary active ingredient of musk. MUS has been shown to treat ischemic stroke by reducing neuronal necrosis through molecular mechanisms that regulate synaptic connections in neurons, shield the blood–brain barrier, and ameliorate neurological impairment due to cerebral ischemia [1,2,3]. Additionally, MUS exhibits efficacy in treating Alzheimer’s disease by protecting APP/PS1 mice from damage to synaptic plasticity and memory [4,5]. MUS also demonstrates a certain inhibitory effect on gastric cancer by regulating miRNA-145 to limit the growth and activity of SGC-7901 and MGC-803 gastric cancer cell lines in vitro conditions. [6,7]. Furthermore, MUS exerts protective effects on the heart by inhibiting oxidative stress and cell apoptosis, thereby treating coronary heart disease [8,9]. However, its volatility, poor water solubility, and low bioavailability limit its development and utilization as a drug.

Metal–organic frameworks (MOFs) are structurally diverse materials composed of inorganic and organic components. Due to their large surface area, tunable structural functionalization, and porosity, MOFs are being developed for various potential applications, such as sensing [10,11,12,13], chromatographic separation [14,15,16], gas storage [17,18], template synthesis [19,20,21], and drug delivery [22,23]. Most MOFs are composed of metals (e.g., Co, Ni, Cr, and Gd), considering potential biological applications [24]. Nonetheless, the compatibility with biological systems and potential harmful effects of MOF are crucial factors to consider when employing them in medical applications [25,26,27]. Consequently, MOFs made up of drug excipients and naturally occurring metal ions play a crucial role in the development of drug delivery systems [28].

Cyclodextrin-based metal–organic frameworks (CD-MOFs) display robust crystallinity, enduring porosity, a substantial specific surface area, and superior biocompatibility [29,30,31]. CD-MOFs possess unique properties such as renewability, edibility, and biodegradability, making them a promising new type of MOF delivery system. The CD-MOF series is broadening through the addition of various metal ions, such as alkali metals (Li+, Na+, K+, Rb+, Cs+) and γ-CD, along with its related compounds. Owing to its significant symmetry, γ-CD features the largest cavity among α-CD and β-CD, rendering γ-CD-MOF especially appealing for drug delivery [32]. Lately, γ-CD-MOF has emerged as an eco-friendly and sustainable framework material. This innovative structure is created by coordinating the secondary surface hydroxyl groups of alternating D-pyranose glucose units with an alkali metal cation, leading to a body-centered cubic arrangement that extends throughout the material [29,30].

CD-MOF’s porous nature is ideal for housing and maintaining guest molecules, making it a prime candidate for storing small molecule substances like curcumin [33], quercetin [34], vitamin A palmitate [35,36], azilsartan [37], etc. CD-MOF not only improves the solubility of the drug but also enhances its stability and bioavailability through its high loading rate and effective encapsulation of drug molecules. Due to the high thermal and mechanical stability inherent in MOFs, CD-MOFs also hold significant potential in the loading and thermal stabilization of unstable compounds. For instance, it has been reported that highly porous CD-MOFs loaded with sucralose provide a sealed microenvironment for the drug, offering numerous interactions with the pharmaceutical compound. The resulting stabilization effect delays the decomposition of sucralose and significantly enhances its thermal stability at high temperatures [38]. The varied locations in the pores of CD-MOF can stabilize anthocyanins via multiple non-covalent intermolecular interactions, thereby improving the stability of anthocyanins to heat or UV irradiation and reducing the loss of their antioxidant properties [39]. Furthermore, in the loading of curcumin, it has been demonstrated that the chemical stability of curcumin in water under alkaline conditions is significantly enhanced [40]. In summary, CD-MOF may represent a promising benign system for the storage and stabilization of some unstable compounds.

In this research, the incorporation of MUS into γ-CD-MOFs produced by the solvent-free technique improves the drug’s stability. A thorough characterization of CD-MOF/MUS was performed, including the molecular interactions with MUS, providing practical references for the study of γ-CD-MOFs in the encapsulation of oily liquids.

## 2. Materials and Methods

### 2.1. Materials

The γ-CD was acquired from Maxdragon Biochemical Co., Ltd. (Guangzhou, China). Muscone (greater than 97 % LOT: J2210589) was offered by Aladdin Biochemical Technology Co., Ltd. (Shanghai, China). Methyl alcohol (LOT: P2800905) and anhydrous alcohol (LOT: P2840825) were provided by Titan Scientific Co., Ltd. (Shanghai, China). KOH (LOT: 20211122) was obtained from Sinopharm Chemical Reagent Co., Ltd. (Shanghai, China). PEG20000 (LOT: C14840977) was sourced from Macklin Biochemical Technology Co., Ltd. (Shanghai, China).RAW264.7 cells (LOT: 240517X201) were obtained from Suzhou Haixing Biological Technology Co., Ltd. (Suzhou, China). RAW264.7 Cell-specific Culture Medium (LOT: 19F13B22) was obtained from Boster Biological Technology Co., Ltd. (Wuhan, China). Other chemicals and reagents were of analytical grade.

### 2.2. Synthesis of CD-MOF and MUS/CD-MOF

The CD-MOF was synthesized via the solvothermal synthesis technique. In brief, 6.49 g of γ-CD (5 mM) and 2.24 g of KOH (40 mM) were weighed and dissolved in 200 mL of ultrapure water. The filtered solution was transferred to a conical flask, and the magnetic stirrer was adjusted to a temperature of 50 °C with a stirring speed of 400 rpm. When the temperature of the reaction medium reached 50 °C, 120 mL of methanol was slowly added, and the reaction mixture was sealed for 20 min. Then, 2.56 g of PEG20000 was added, and the reaction continued for 10 min. After the reaction was complete, the mixture was refrigerated overnight to allow for crystal formation. The obtained crystals were washed multiple times with ethanol and subsequently vacuum-dried at 60 °C for 5 h to obtain CD-MOF crystals [36,41].

The loading of MUS was conducted via 2 strategies, namely, solvent incubation and solvent-free methods. Briefly, for the solvent incubation method, 18.4 mg of MUS was dissolved in 10 mL anhydrous ethanol with the help of ultrasonic treatment (KQ-500DE, Kunshan Ultrasonic Instruments Co., Ltd., Kunshan, China) at 500 W for 5 min to assist dissolution. Then, 100 mg of CD-MOF was added to the solution, and the mixture was stirred at 40 °C and 400 rpm using a magnetic stirrer for 12 h (CD-MOF to MUS molar ratio = 1:1). After the reaction, the resulting particles were centrifuged and washed three times with ethanol, followed by vacuum drying for 6 h to obtain the CD-MOF/MUS particles. The experiment was repeated by varying the CD-MOF to MUS molar ratios to 1:5, 1:10, and 1:20 while keeping the temperature, rotation speed, and reaction time constant. Additionally, the reaction temperature was increased to 50 °C, and the above procedure was repeated to investigate the effect of temperature and CD-MOF to MUS molar ratio on the loading of MUS by the solvent method. Each set of experiments was conducted in triplicates.

For the solvent-free method, 1 mL of MUS was directly mixed with 100 mg of CD-MOF, and the mixture was stirred at 50 °C and 400 rpm on a magnetic stirrer for 12 h. After the reaction, the particles obtained were centrifuged and washed three times with ethanol, then dried to obtain the CD-MOF/MUS particles. The effect of temperature on the loading of MUS using the solvent-free method was investigated by changing the reaction temperature to 60 °C and 80 °C while keeping the CD-MOF to MUS ratio, rotation speed, and reaction time constant. This experimental setup allowed for the assessment of temperature’s influence on the solvent-free method for loading MUS.

### 2.3. Establishment of MUS Content Detection Method

Pure MUS (12.5 mg) was dissolved in ethyl acetate to a final volume of 25 mL. This solution was then used to prepare standard solutions of 50 μg/mL, 100 μg/mL, 150 μg/mL, 200 μg/mL, 450 μg/mL, and 500 μg/mL for GC-MS detection. For drug-loading quantification, CD-MOF/MUSs (5 mg) were accurately weighed. Then, 50% DMSO in water was added to the sample and dissolved with the aid of ultrasonication. Ethyl acetate 2 mL was then added for extraction and vortexed for 1 min. The mixture was allowed to stand for phase separation, and then the upper layer was taken and filtered for GC-MS detection.

### 2.4. GC-MS Analysis

Quantitative analysis of MUS-loaded samples was performed using a high-sensitivity GC-MS system consisting of an Agilent 7890A GC (Agilent Technologies, Santa Clara, CA, USA) and an Agilent 5975C (Agilent Technologies, Santa Clara, CA, USA) inert mass spectrometer. The chromatographic conditions are as follows: chromatographic column, Agilent 123-1334 DB-624 (Agilent Technologies, Santa Clara, CA, USA, 30 m × 320 μm × 1.8 μm); injection port temperature, 200 °C, using a split injection mode with a split ratio of 5:1; carrier gas, high-purity helium, in constant linear velocity mode, with a flow rate of 10 mL/min; and injection volume, 1 μL. The temperature program is as follows: initial temperature 100 °C (hold for 1 min), ramp to 200 °C at 12 °C/min (hold for 3 min), and then ramp to 280 °C at 20 °C/min (hold for 8 min). Ion source: EI ion source; electron energy: 70 eV; ion source temperature: 230 °C; quadrupole temperature: 150 °C; tuning mode: automatic tuning; mass scanning mode: full scan; scanning range: 30–650 amu; and threshold: 100 [42,43].

The levels of MUS were determined using a linear calibration curve created within a concentration range of 50 to 500 μg/mL. The standard curve for MUS was developed employing the least squares method, utilizing a weighting of 1/x^2^. The typical standard curve equation for MUS was R = 135,225C + 6,000,000 (the standard curve of MUS is shown in Supplementary File Figure S1), where R is the peak area ratio of MUS and C is the concentration of MUS (r^2^ = 0.9994).

### 2.5. Characterizations

#### 2.5.1. Scanning Electron Microscopy (SEM)

The surface morphology of CD-MOF and CD-MOF/MUS was observed by scanning electron microscope (TESCAN MIRA LMS, TESCAN CHINA, Ltd., Shanghai, China), and elemental mapping analysis was conducted.

#### 2.5.2. X-Ray Single Crystal Diffraction

X-ray single crystal diffractometer (XtaLAB PRO II, Rigaku Corporation, Tokyo, Japan) was compressed into a uniform thin disc and subsequently mounted onto the sample stage of the X-ray diffractometer for analysis.

#### 2.5.3. Fourier-Transform Infrared Spectroscopy

Fourier-transform infrared spectroscopy (FTIR) was used to characterize CD-MOF, MUS, CD-MOF/MUS, and the physical combination of CD-MOF and MUS. The sample was thoroughly mixed with potassium bromide and compressed into a tablet.

#### 2.5.4. Thermogravimetric-Differential Scanning Calorimetry (TG-DSC)

The thermal stability of MUS, CD-MOF, and CD-MOF/MUS was analyzed using a TG-DSC coupled instrument (TG-DSC, Netzsch STA 449 F3, Netzsch Gerätebau GmbH, Selb, Germany) in the temperature range of 30 °C to 615 °C (heating rate of 20 °C/min).

#### 2.5.5. Nitrogen Adsorption Isotherm

The specific surface area and pore volume of the samples were assessed using an automatic surface area and porosity analyzer (BET, Micromeritics ASAP 2460, Micromeritics Instrument Corporation, Norcross, GA, USA). Prior to gas adsorption, CD-MOF and CD-MOF/MUS were degassed at 60 °C for 12 h to eliminate remaining solvent and moisture, and the adsorption of N_2_ was tested at liquid nitrogen temperature (77 K).

### 2.6. Solubility and In Vitro Release Studies

To prepare supersaturated solutions of MUS and CD-MOF/MUS (equivalent to the MUS content), three sets of solutions were prepared. These solutions were then placed in a constant temperature shaker at 37 °C, with shaking set to 150 rpm/min for 24 h. After the incubation, 2 mL of the solution was taken, followed by the addition of 1 mL of ethyl acetate for extraction. The mixture was vortexed for 2 min, then centrifuged at 6000 rpm for 10 min. The upper phase was collected and analyzed using GC-MS to determine the MUS concentration.

Accurately, 1 mg of MUS and an equal amount of CD-MOF/MUS were dispersed in 5 mL of 0.5% Tween 80 (*v*/*v*) phosphate-buffered saline (PBS) with pH 7.4. The mixture was then transferred into a dialysis bag (molecular weight cutoff: 3.5 kDa). The dialysis bag was placed in 25 mL of 0.5% Tween 80 (*v*/*v*) PBS solution. The system was shaken at 150 rpm/min and maintained at 37.5 °C. At designated time points (1, 2, 4, 6, 8, 12, 24, and 48 h), 2 mL of the release medium was collected from each group and replenished with an equal volume of fresh medium. The collected release medium was extracted with 1 mL of ethyl acetate and vortexed for 5 min, followed by centrifugation at 6000 rpm for 10 min. The upper phase was collected and analyzed using GC-MS to determine the cumulative release percentage of MUS. Three parallel samples were used for each group.

### 2.7. Cell Viability Assays

RAW264.7 cells (mouse monocyte macrophage leukemia cells) were used for the evaluation of cell viability. The cells were plated in a 96-well plate at a density of 10,000 cells per well and incubated in a humidified environment at 37 °C, with an atmosphere comprising 95% air and 5% CO_2_, for a duration of 24 h. Following this incubation, solutions of MUS and CD-MOF/MUS were prepared at concentrations ranging from 2.5 to 40 μg/mL (corresponding to the MUS concentration levels), while CD-MOF solutions were prepared at concentrations between 100 and 1000 μg/mL in a specialized culture medium. These solutions were added to the respective wells, and the cells were incubated for an additional 24 h (n = 6). After the incubation, 10 μL of CCK8 reagent was added to each well, and the absorbance of the cells at a reference wavelength of 450 nm was measured using a microplate reader (Multiskan GO, Thermo Fisher, Waltham, MA, USA). Untreated cells were set as the control group, and the specialized culture medium was designated as the blank group.

(1) $$\mathrm{Cell}\;\mathrm{viability}\;\left(\%\right)=\frac{{\mathrm{OD}}_{\mathrm{Drug}}-{\mathrm{OD}}_{\mathrm{Blank}}}{{\mathrm{OD}}_{\mathrm{Control}}-{\mathrm{OD}}_{\mathrm{Blank}}}\text{×}100$$

where OD_Drug_ indicated the absorbance of the sample, OD_Blank_ indicated the absorbance of the blank, and OD_Control_ indicated the absorbance of the control sample.

The cell viability of MUS, CD-MOF/MUS, and CD-MOF in RAW264.7 cells was analyzed using GraphPad Prism 9.5 (GraphPad Software, Inc., San Diego, CA, USA).

### 2.8. Stability Assessment

To evaluate the stability of the prepared CD-MOF/MUS, we assessed the thermal and photostability. The thermal stability of MUS was examined at 50 °C, 25 °C, and 4 °C for a period of 30 days. Similarly, the photostability was observed by light exposure over 7 days, 14 days, and 30 days. The specific procedure involved transferring CD-MOF/MUS particles and MUS into 10 mL EP tubes, which were then preserved at the various conditions. Samples of both CD-MOF/MUS and MUS were taken at 0, 7, 14, and 30 days for assessment. Each experiment underwent three repetitions. The formula employed to determine the remaining MUS rate is as follows:

(2) $$\mathrm{The}\;\mathrm{residual}\;\mathrm{rate}\;\mathrm{of}\;\mathrm{MUS}\left(\%\right)=C_{t}/C_{0}\text{ × }100$$

where C_0_ indicated the original content of MUS; C_t_ denoted the content of MUS at various sampling intervals.

### 2.9. Molecular Dynamics Simulation

The crystal structure of CD-MOF was obtained from the single-crystal data of CD-MOF-1 CCDC. The molecular structure of MUS was obtained from PubChem. Energy minimization and molecular dynamics simulations were conducted using Amber 12, with Amber Tools preparing the initial setup. Force field parameters for γ-CD were sourced from GLYCAM-Web. Partial atomic charges for MUS were computed using Gaussian 09. AutoDock Vina 1.1.2 facilitated the initial positioning of MUS. The MUS model underwent energy minimization before docking with the CD-MOF structure. The lowest-energy docking result was selected for further optimization [37]. During minimization, models were placed in a vacuum, followed by a 20 ns molecular dynamics simulation. Parameters included a nonbond cutoff of 18.5 Å, a spline width of 1.0 Å, and a buffer width of 0.5 Å. Docking employed a Lamarckian Genetic Algorithm (LGA) and grid-based energy evaluation, precomputing grid maps for interatomic potentials. A 30 Å × 30 Å × 30 Å grid with 0.375 Å spacing encompassed the CD-MOF model. AutoDock Vina (The Scripps Research Institute, La Jolla, CA, USA, version 1.1.2) calculated atomic partial charges using the Gasteiger–Marsili method. To assess the thermodynamics of the system, the Amber14sb_parmbsc1 all-atom force field was employed. The weak interactions between CD and MUS were calculated using the independent gradient model (IGM) based on Multiwfn as reported by Zhao et al. [34].

### 2.10. Statistical Analysis

All data are presented as mean ± standard deviation (SD), with error bars representing the SD. Statistical analyses were performed using GraphPad Prism version 9.5.0 (GraphPad Software, Inc., San Diego, CA, USA). For comparisons among multiple groups, one-way ANOVA, followed by Tukey’s multiple comparisons test, was conducted to assess statistical significance. Significance levels are indicated as follows: * *p* < 0.05, ** *p* < 0.01, *** *p* = 0.0001, **** *p* < 0.0001 (adjusted *p*-values from multiple comparisons).

## 3. Results and Discussion

### 3.1. Analysis of MUS Load Results Using Different Methods

**Figure 2:**
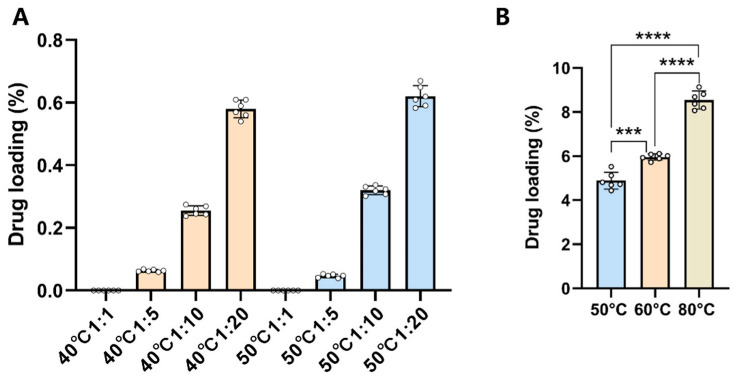
Drug-loading optimization process. (**A**) Drug-loading capacity chart for different groups using the solvent method, (**B**) drug-loading chart for different groups using the solvent-free method (n = 6, mean ± SD, *** *p* = 0.0001, **** *p* < 0.0001).

The results of the solvent-based drug-loading method revealed that the loading efficiency for each group was below 0.8% (Figure 2A). However, increasing the temperature demonstrated a modest enhancing effect on loading efficiency. In contrast, the solvent-free method yielded significantly higher loading efficiencies, reaching 8.5% ± 0.4 at 80 °C (Figure 2B). Notably, the loading efficiency exhibited a positive correlation with temperature. Further optimization of the solvent-based method at 80 °C with a 12 h reaction time resulted in a substantial improvement in MUS loading, achieving a loading rate of 10.6% ± 0.7 in a scaled-up experiment.

### 3.2. Characterization and Mechanism Investigation

**Figure 3:**
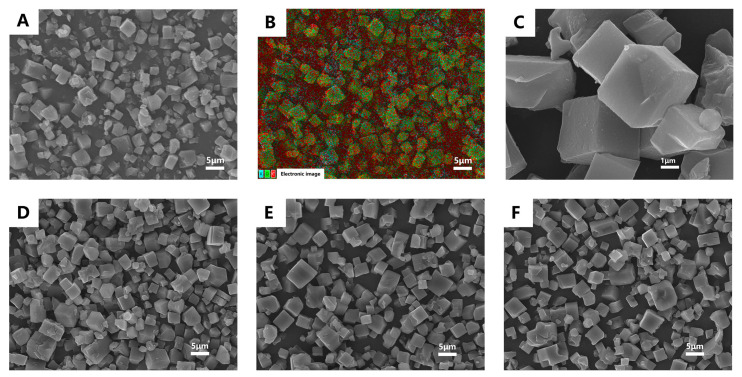
CD-MOF micromorphology. (**A**) SEM of CD-MOF, (**B**) EDS spectroscopy of CD-MOF, (**C**) SEM of CD-MOF, the scale bar was 1 μm, (**D**) SEM of CD-MOF/MUS at 50 °C, (**E**) SEM of CD-MOF/MUS at 60 °C, (**F**) SEM of CD-MOF/MUS at 80 °C.

The SEM images show that the CD-MOF particles are uniform cubic particles with an average size of 4–8 μm. The EDS (Energy-dispersive X-ray Spectroscopy) spectra indicate the elemental makeup of various CD-MOF materials (Figure 3A–C), and the results show the presence of three elements, with carbon and oxygen originating from γ-cyclodextrin, while potassium comes from the metal ions, further confirming the successful preparation of CD-MOF. Additionally, after loading MUS under different temperature conditions, the shape of the particles is still maintained (Figure 3E,F), indicating that the temperature during the MUS loading process does not damage the crystal structure of CD-MOF.

**Figure 4:**
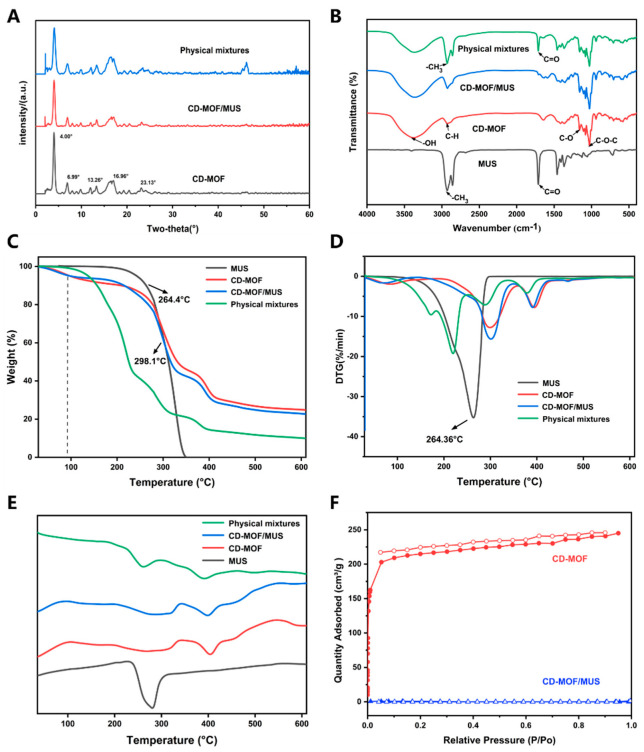
Characterizations. (**A**) XRD patterns, (**B**) FT−IR spectra, (**C**–**E**) TGA, DTG, and DSC profiles, (**F**) N_2_ adsorption−desorption isotherms.

The X-ray characteristic diffraction peaks of CD-MOF are at 4°, 6.99°, 13.26°, 16.96°, and 23.13° (Figure 4A), which are consistent with reports in the literature [44]. In the drug-loaded group, these peaks remain, indicating that the crystallinity of CD-MOF remains intact following the incorporation of MUS into CD-MOF. FTIR results show that the broad peak around 3378 cm^−1^ is due to the -OH stretching vibration within the glucose ring, while the peak at 2922 cm^−1^ corresponds to the stretching vibrations of C-H bonds in CH_2_ and CH groups. The bending band of symmetrical water located in the γ-CD macrocyclic cavity is clearly observed at 1648 cm^−1^, and the peaks around 1158 cm^−1^ and 1025 cm^−1^ correspond to C-O stretching and C-O-C bending vibrations. The stretching absorption of the -CH3 group in MUS appears around 2927 cm^−1^, and the stretching vibration of C=O is at 1713 cm^−1^. The characteristic peaks of CD-MOF/MUS (2927 cm^−1^, 1713 cm^−1^) are almost not visible (Figure 4B), but the characteristic peaks of MUS in the physical mixture still exist, indicating that MUS is effectively loaded into CD-MOF, rather than just being adsorbed on the surface.

The thermal stability of the material was evaluated using thermogravimetric analysis (TGA) and derivative thermogravimetry (DTG), as shown in Figure 4D,E. A significant weight loss between 50 °C and 120 °C was observed, which is primarily attributed to entrapped moisture or residual solvent from the cavities of the CD-MOF carrier during the washing and drying process [45]. A subsequent phase of degradation between 250 °C and 350 °C indicates the breakdown of both the crystalline structure and organic components [46,47]. CD-MOF exhibited a clear degradation peak at 298.77 °C, attributed to the rupture of weak metal–ligand coordination bonds and the collapse of its distinctive framework [48]. The results reveal a shift in the degradation pattern of MUS after its incorporation into the CD-MOF framework. Furthermore, differential scanning calorimetry (DSC) analysis showed a prominent endothermic peak for MUS at 275.29 °C, consistent with its inherent thermal properties. The characteristic peaks of MUS are still present in the simple physical mixture, as shown in Figure 4E (further information is provided in the Supplementary File). In contrast, the CD-MOF/MUS drug-loaded system did not exhibit any endothermic or exothermic peaks at this temperature, suggesting that MUS was successfully incorporated within the CD-MOF framework after the loading process. This observation implies the presence of intermolecular interactions between the drug and the carrier. Importantly, the maximum thermal degradation peak of MUS (264.36 °C) shifted to a lower temperature after being incorporated into the CD-MOF, providing additional evidence for the effective loading of MUS into the framework.

The Brunauer–Emmett–Teller (BET) technique was utilized to gauge the specific surface area and porosity, as depicted in Figure 3F. Using the same analysis, we delved into the permanent porosities of the CD-MOF both pre- and post-drug incorporation. The nitrogen isotherm exhibited a sharp increase in adsorption at low pressures. However, upon the addition of MUS, the nitrogen adsorption substantially dropped, approaching nil.

### 3.3. Solubility and In Vitro Release Results

In the solubility experiment, the concentration of pure MUS in water was found to be 0.21 ± 0.03 μg/mL. After loading into CD-MOF, the solubility of MUS in water increased to 5.1 ± 0.6 μg/mL, which is 24 folds more than pure MUS, indicating that CD-MOF significantly enhances the solubility of MUS. This CD-MOF system is designed to undergo controlled dissociation in aqueous media, thereby releasing encapsulated drugs and improving MUS solubility profile. While this property precludes long-term stability in biological fluids, it is mechanistically essential for the carrier’s drug release function [49]. Furthermore, the 24-fold solubility enhancement positions CD-MOF/MUS as a viable system for further development. In research studies (animal models), the MUS concentration for intragastric administration in animals generally ranges from 1 mg/kg to 10 mg/kg, but human equivalents would be much lower, and, therefore, the CD-MOF/MUS loading percent is sufficient for further investigation [50,51].

**Figure 5:**
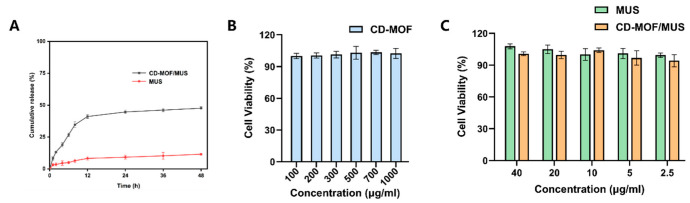
In vitro release and cell viability assay. (**A**) In vitro release curve, (**B**) inhibition rates of cell growth in RAW264.7 due to CD-MOF, (**C**) MUS compared to CD-MOF/MUS (n = 6, mean ± SD).

The release curve (Figure 5A) reveals that the solubility of pure MUS in the release medium is extremely low, while CD-MOF/MUS shows a significantly higher release profile, with 44.7% ± 1.3 released after 24 h, compared to just 9.2% ± 1.5 for pure MUS. A comparison of the release rates between MUS and CD-MOF/MUS shows that the release rate of CD-MOF/MUS is remarkably higher than that of the free drug. Furthermore, the release profile did not show a burst release, which highlights the successful incorporation within the CD-MOF cavity. The CD-MOF/MUS particles (4–8 μm) are optimally sized for oral administration, as demonstrated by prior studies with CD-MOF carriers [37,41,52]. For oral delivery, the porous γ-CD-MOF structure protects MUS from gastric degradation and facilitates sustained release in the intestine (44.7% over 24 h in PBS pH 7.4), promoting absorption in the neutral intestine, where its solubility-enhanced formulation promotes absorption (Figure 5A). CD-MOF drug release in aqueous media involves hydrolysis of metal–ligand bonds and ion exchange destabilizing the framework, controlled diffusion through porous channels/swelling, and disruption of host–guest interactions as water displaces drugs from CD cavities via competitive binding or concentration gradients [31,32]. This aligns with reports showing CD-MOF enhances the bioavailability of hydrophobic drugs like azilsartan [37] and quercetin [47] via mucosal adhesion and controlled GIT release, highlighting the advantages of the CD-MOF carrier for drug delivery.

### 3.4. Cell Viability Assay

The biocompatibility of CD-MOF was assessed with RAW264.7 cells, and the findings indicated that there was no notable inhibition of cell growth across the tested concentration range of 100–1000 μg/mL. Notably, cell viability remained stable at 100%, indicating that CD-MOF possesses excellent biocompatibility and potential as a drug carrier (Figure 5B). Furthermore, both the MUS group and the CD-MOF/MUS drug-loaded group demonstrated no significant growth inhibition on cells within the 2.5–40 μg/mL concentration span (Figure 5C). This suggests that the biocompatibility of CD-MOF is maintained even after loading with MUS, emphasizing its promise as a secure and efficient drug delivery method. The use of RAW264.7 cells was particularly advantageous for this study due to their rapid proliferation and suitability for high-throughput screening during formulation optimization. However, while these macrophage-like cells provide valuable preliminary safety data, they cannot fully replicate the epithelial barriers encountered in oral delivery. Future studies employing gastrointestinal models (e.g., Caco-2 cells) would be essential to specifically evaluate the formulation’s performance for oral administration routes.

### 3.5. Stability Analysis

**Figure 6:**
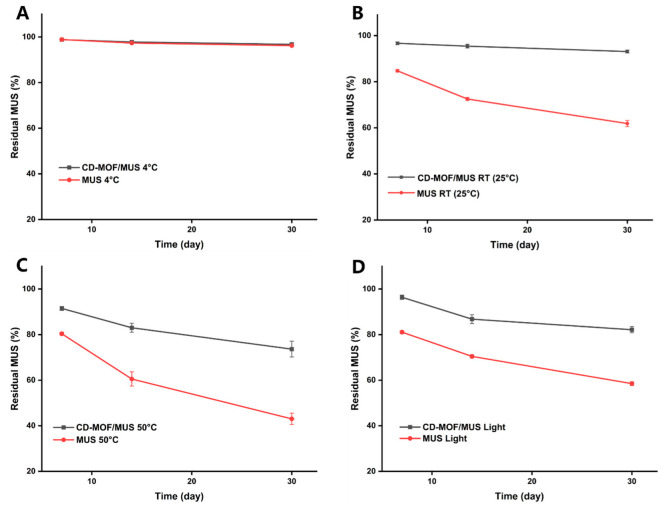
Investigation of the stability. (**A**) Comparison of MUS and CD-MOF/MUS under 4 °C conditions, (**B**) comparison of MUS and CD-MOF/MUS under room temperature conditions, (**C**) comparison of MUS and CD-MOF/MUS under 50 °C conditions, (**D**) comparison of MUS and CD-MOF/MUS under light conditions.

The stability test results demonstrate that storing MUS at 4 °C has minimal impact on its content over time. After 7, 14, and 30 days, the residual amounts of MUS in the MUS group were 98.9% ± 0.3, 97.3% ± 0.4, and 96.2% ± 0.4, respectively. Similarly, the CD-MOF/MUS group showed residual amounts of 98.8% ± 0.3, 97.8% ± 0.6, and 96.7% ± 0.8, respectively. Both groups exhibited high residual amounts and similar stability profiles (Figure 6A).

At room temperature, the MUS group demonstrated a gradual decrease in residual amounts, with values of 84.7% ± 0.5, 72.5% ± 0.5, and 61.9% ± 1.1 after 7, 14, and 30 days, respectively. In contrast, the CD-MOF/MUS group maintained significantly higher residual levels, showing 96.7% ± 0.5, 95.4% ± 0.7, and 93.1% ± 0.5 after the same periods. This indicates that the stability of MUS was significantly enhanced by its inclusion in the CD-MOF carrier system (Figure 6B).

Under conditions of 50 °C, the residual amounts of MUS in the MUS group were 80.4% ± 0.6, 60.5% ± 2.6, and 43.0% ± 2.0 after 7, 14, and 30 days, respectively. In comparison, the CD-MOF/MUS group exhibited residual amounts of 91.5% ± 0.7, 83.0% ± 1.6, and 73.6% ± 2.8, respectively. These results suggest that while elevated temperatures still impact the stability of MUS, the CD-MOF significantly improves its stability under these conditions (Figure 6C).

When exposed to light, the residual amounts of MUS in the MUS group were 81.1% ± 0.5, 70.4% ± 0.6, and 58.5% ± 0.7 after 7, 14, and 30 days, respectively. The CD-MOF/MUS group showed significantly higher residual amounts of 96.4% ± 0.8, 86.8% ± 1.6, and 82.1% ± 1.1, indicating that the inclusion of CD-MOF mitigates the degradation caused by light exposure (Figure 6D).

The stability results across all four conditions (4 °C, room temperature, 50 °C, and light exposure) collectively indicate that loading MUS into CD-MOF enhances its overall stability. This improvement can be attributed to the protective effects of the MOF matrix, which shields the drug from environmental stressors such as temperature and light. These findings highlight the potential of CD-MOF as a stable and effective drug delivery system for MUS. Nevertheless, further studies are necessary to optimize storage conditions and evaluate the long-term stability of the CD-MOF/MUS formulation.

### 3.6. Molecular Dynamics Simulation Findings

**Figure 7:**
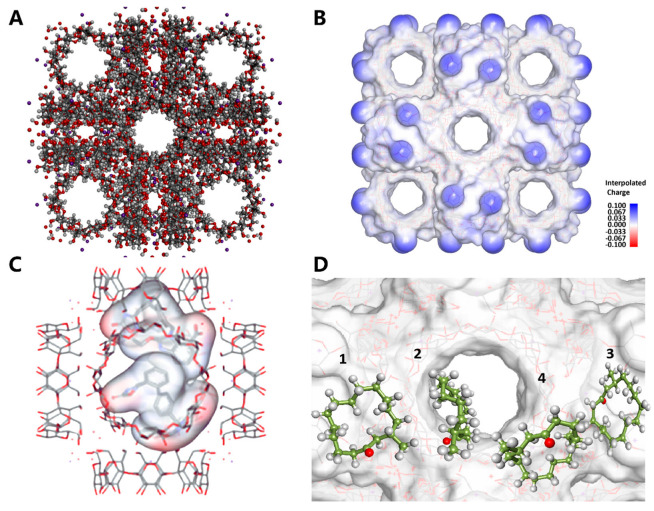
Molecular dynamics simulation. (**A**) Ball and stick representation of CD-MOF, (**B**) charge distribution in CD-MOF, (**C**) distribution of two molecules of MUS in CD-MOF cavity, (**D**) weak interaction between MUS and γ-CD on entry and exit.

To elucidate the MUS loading process in CD-MOF, molecular docking simulations were deployed to probe the binding interactions between CD-MOF and MUS. The analysis revealed that non-covalent forces and auxiliary factors govern the interaction of free energy. We performed energy optimization and molecular dynamics calculations using low-energy parameters to simulate the behavior of MUS within CD-MOF (Figure 7A,B) [37,53]. The results indicated a preferential occupation of MUS molecules in the large spherical cages of CD-MOF, measuring 1.7 nm in diameter, as opposed to the dicyclodextrin cavity, as illustrated in Figure 7C [52]. Notably, the simulation results demonstrated that muscone molecules can coexist within the cage-like confines of CD-MOF, particularly at elevated concentrations or prolonged loading times exceeding 10 h. Moreover, experimental data confirmed that a 1:10 molar ratio of muscone to γ-CD yields optimal loading capacities.

The electrostatic potential analysis further revealed that resonant potential influences the system’s thermodynamic behavior, prompting the exploration of high free energy regions [34]. By accounting for this influence, we calculated the free energy (ΔG) and obtained the system′s quantitative thermodynamic description, as shown in Figure 7D. The result showed that when a muscone molecule approaches CD-MOF, ΔG decreases to −4.9 kcal/mol because of the weak interactions, revealing spontaneous adsorption of muscone onto γ-CD. Our findings suggest that muscone molecules exhibit spontaneous affinity for γ-CD rings, necessitating additional energy of approximately 6.0 kcal/mol for escape or migration between CD-MOF unit cells.

## 4. Conclusions

This study successfully synthesized γ-CD-MOF as a drug carrier using a solvent-free method, demonstrating its innovative application in oral drug delivery systems. Compared to synthetic MOFs, CD-MOF exhibits excellent biocompatibility with no cytotoxicity and effectively loads MUS, achieving an optimal drug-loading rate of 10.6% ± 0.7. The system significantly improves MUS solubility by 24-fold while stabilizing the drug and reducing volatility, key advantages for controlled release. While CD-MOF/MUS significantly improves MUS stability and solubility, its behavior in physiological media (e.g., gastric fluid, plasma) requires further validation. Prior studies indicate that CD-MOFs undergo degradation, which could impact drug release kinetics. Future work will assess stability in simulated biological fluids (SGF/SIF) and albumin solutions, alongside surface modifications to enhance physiological compatibility.

Various characterization techniques, including XRD, SEM, TG-DSC, FTIR, and molecular docking, were employed to systematically validate the enhancement of MUS stability by γ-CD-MOF. This study is the first to demonstrate the significant advantages of this carrier system in improving MUS stability, reducing volatility, and enhancing solubility. These findings not only provide new insights into the design of MUS delivery systems but also lay the foundation for future pharmaceutical applications. Overall, as an innovative drug carrier, γ-CD-MOF holds significant potential in drug delivery systems, particularly in improving drug stability, enhancing solubility, and enabling controlled release, with promising prospects for future development.

## Data Availability

The original contributions presented in this study are included in the article/Supplementary Material. Further inquiries can be directed to the corresponding author.

## Supplementary Materials

The following supporting information can be downloaded at: https://www.mdpi.com/article/10.3390/pharmaceutics17040497/s1, Figure S1. Standard Curve of MUS. Figure S2. TG-DSC Analysis Chart of CD-MOF. Figure S3. TG-DSC Analysis Chart of MUS. Figure S4. TG-DSC Analysis Chart of CD-MOF/MUS. Figure S5. TG-DSC Analysis Chart of Physical Mixture.
