# Supplementary material for: Preparation and Characterization of Muscone Oil-Based Cyclodextrin Metal–Organic Frameworks: Molecular Dynamics Simulations and Stability Evaluation
Source: Pharmaceutics. 2025 Apr 9;17(4):497. doi: 10.3390/pharmaceutics17040497 (PMC12030149; doi:10.3390/pharmaceutics17040497)
Supplement: Supplementary file 1 [file pharmaceutics-17-00497-s001.zip › pharmaceutics-3531984-supplementary.pdf]

Supplementary File

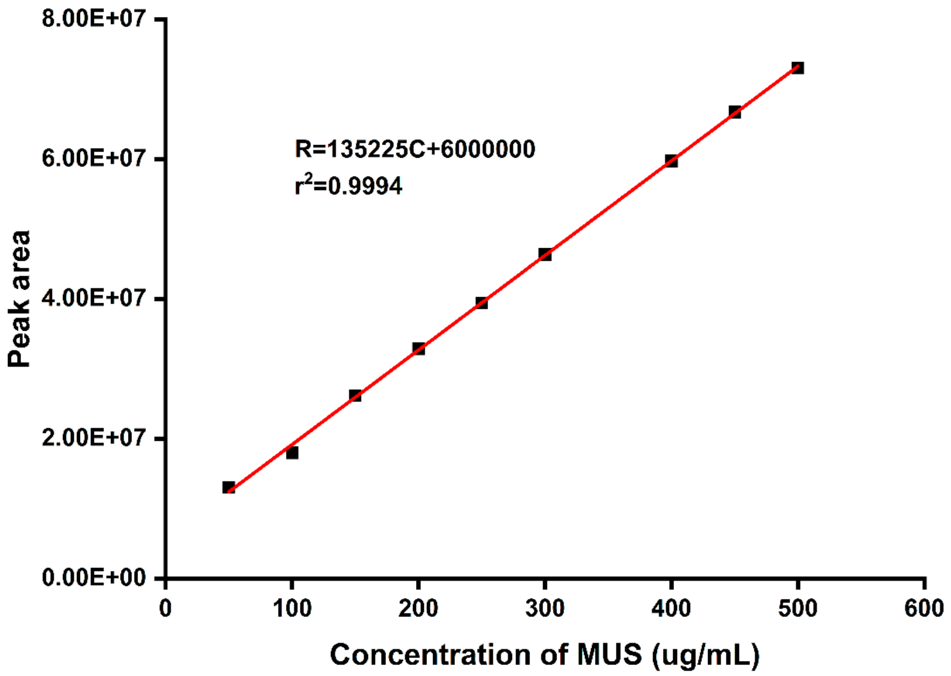

Figure S1. Standard Curve of MUS.

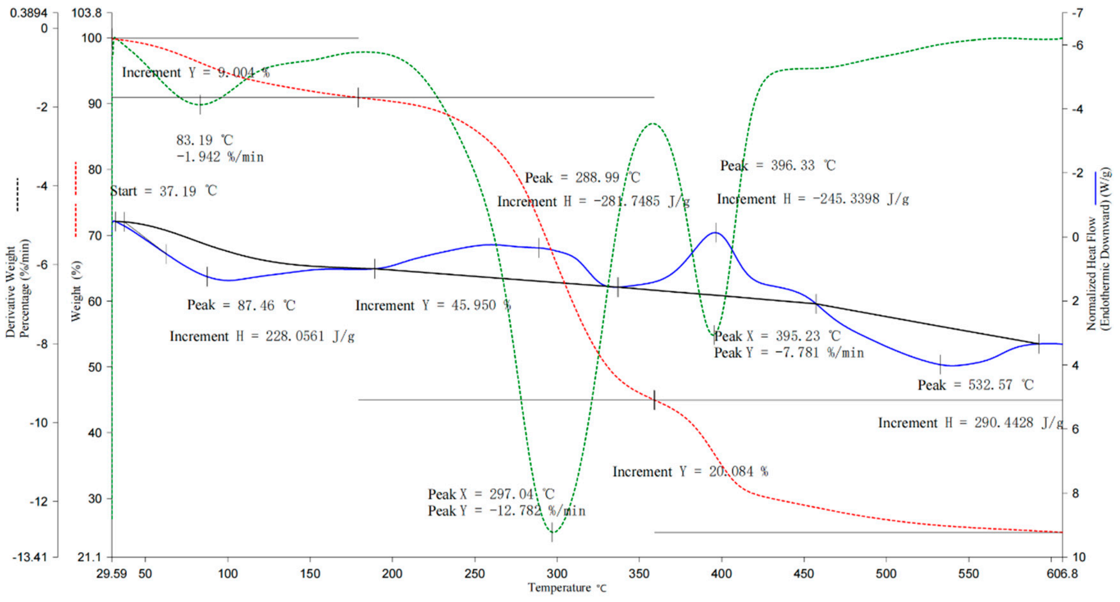

Figure S2. TG-DSC Analysis Chart of CD-MOF.

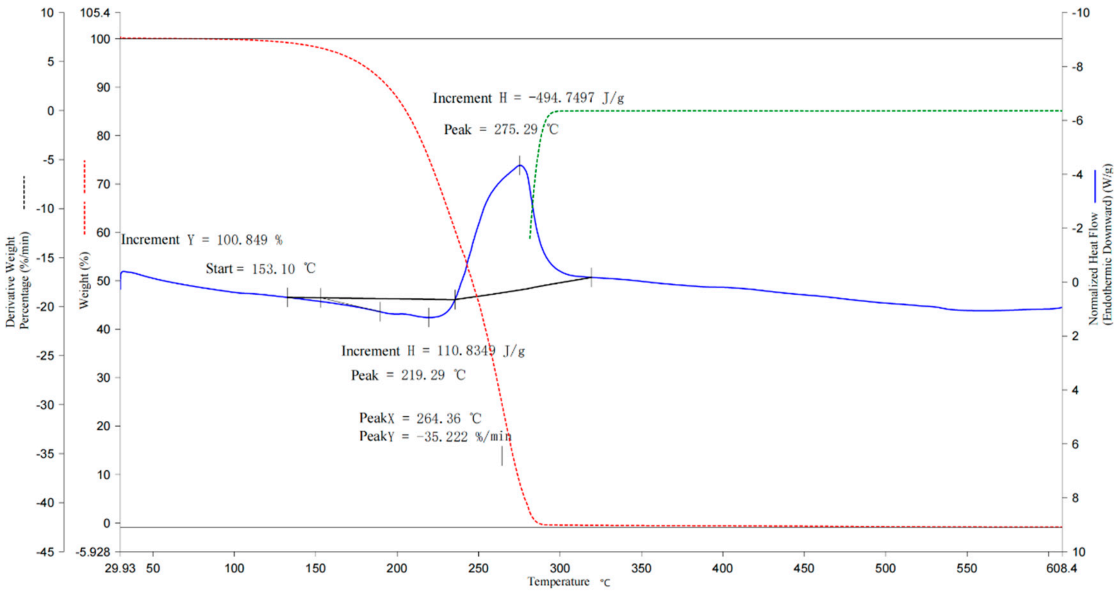

Figure S3. TG-DSC Analysis Chart of MUS.

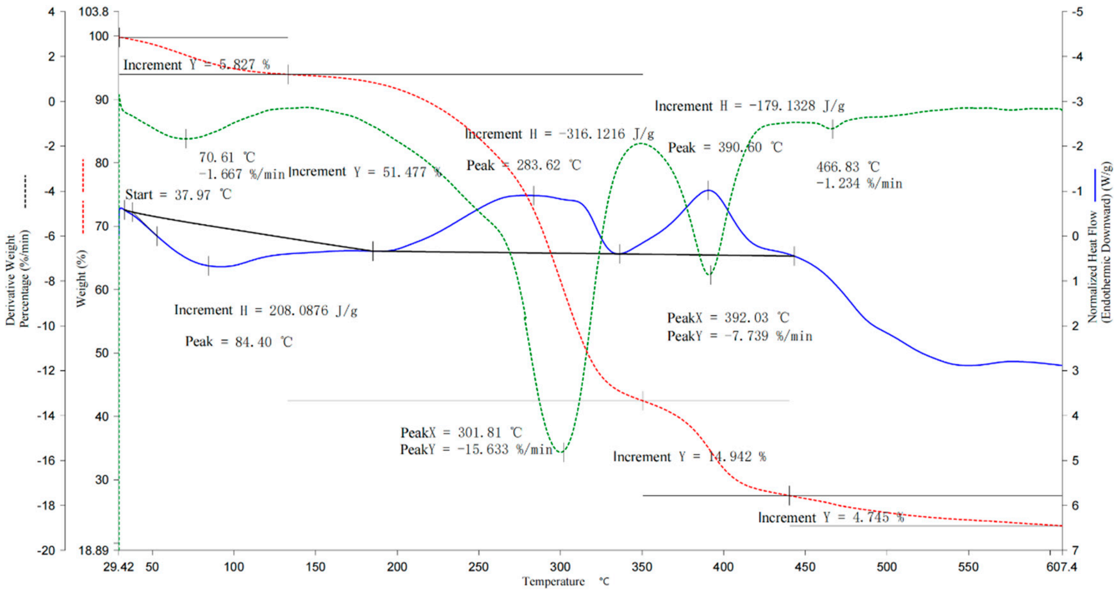

Figure S4. TG-DSC Analysis Chart of CD-MOF/MUS.

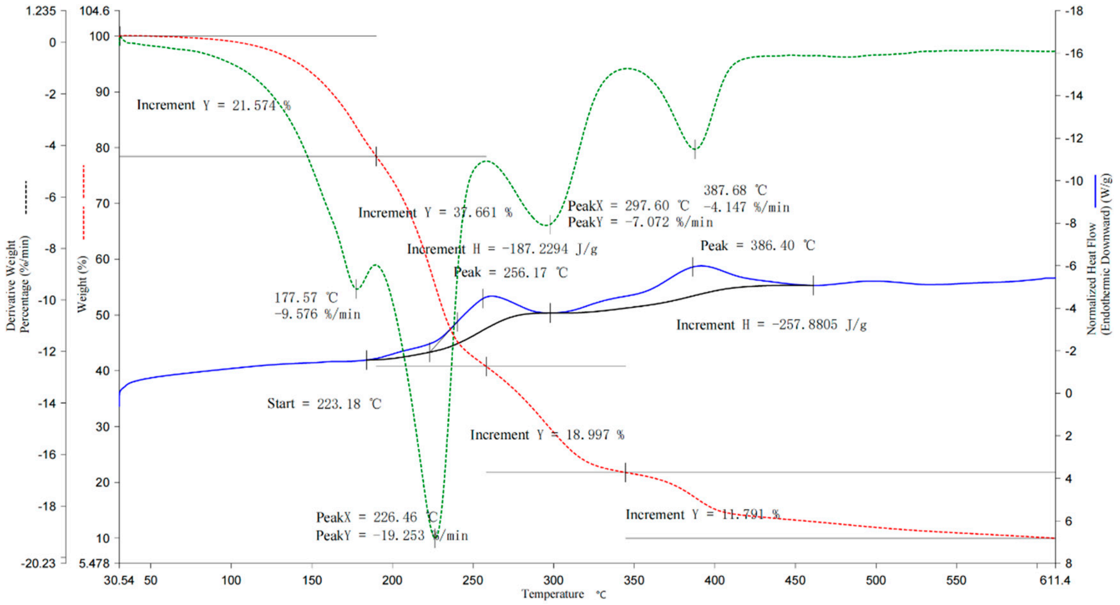

Figure S5. TG-DSC Analysis Chart of Physical Mixture.
